# Disability-based disparities under universal health coverage among chronically ill adults during the COVID-19 pandemic in Indonesia: an interrupted time series analysis

**DOI:** 10.1080/16549716.2025.2581946

**Published:** 2025-11-07

**Authors:** Nuzulul Kusuma Putri, Robeth Jabbar Syahansyah

**Affiliations:** a International Health and Sustainable Development Department, Tulane University Celia Scott Weatherhead School of Public Health and Tropical Medicine, New Orleans, Louisiana, USA; b Health Policy and Administration Department, Faculty of Public Health, Universitas Airlangga, Surabaya, Indonesia; c Research Group for Health and Well-Being of Women and Children, Universitas Airlangga, Surabaya, Indonesia; d Directorate of Medical Services, Dr. Soetomo General Academic Hospital, Surabaya, Indonesia

**Keywords:** Quality of Care for Chronic Conditions, Health system resilience, Gender equality, social insurance, continuity of care, SDG 5: Gender equality

## Abstract

**Background:**

People with disabilities (PWD) face persistent barriers to healthcare, often exacerbated during public health emergencies. In Indonesia, 10.8% of adults with disabilities have chronic disease, yet how the National Health Insurance (JKN) addresses disparities remains unclear.

**Objective:**

To assess the COVID-19 pandemic’s impact on disability-based disparities in chronic healthcare utilization under JKN, and whether these were modified by gender or geographic disadvantage.

**Methods:**

A panel-based interrupted time series analysis using generalized estimating equations was conducted on the JKN sample dataset (1% of national enrollees). The sample included 108,762 adults aged 19–65 with chronic conditions, with monthly primary care records from September 2019 to August 2020. Outcomes were preventive and curative visits, modeled by disability status, gender, and residence district.

**Results:**

The sample averaged 48.3 years, 61.2% were women, 15.5% had a disability, and 1.2% lived in underdeveloped districts. Preventive visits declined 25.6% and curative visits 40.5% in the first two months of the pandemic. PWD had higher curative care use (IRR = 1.05; 95% CI: 1.02–1.07), but women with disabilities had fewer curative visits than men with disabilities (IRR = 0.92; 95% CI: 0.86–0.98). Residence in underdeveloped districts reduced preventive (IRR = 0.34; 95% CI: 0.19–0.60) and curative (IRR = 0.83; 95% CI: 0.70–0.99) visits.

**Conclusions:**

The pandemic worsened disparities. Gender compounded disability-related inequities in curative use, while geographic disadvantage limited access for all. Future research should examine longer-term trends. Integrating disability- and gender-inclusivetuy approaches is essential for equitable UHC during and beyond crises.

## Paper Context

● **Main findings**: During the COVID-19 pandemic, individuals with disabilities had higher curative care utilization, but women with disabilities faced significantly lower access compared to men. Geographic disadvantage also reduced access for all groups regardless of disability status.

● **Added knowledge**: Using panel-based interrupted time series analysis, this study is the first to quantify disability-based disparities in healthcare utilization underutilization under Indonesia’s National Health Insurance (JKN) during a public health crisis.

● **Global health impact for policy and action**: Disability-inclusive and gender-responsive reforms are essential to ensure universal health coverage systems like JKN equitably serve structurally marginalized populations, particularly during systemic disruption.

## Background

Universal health coverage (UHC) has become a central goal of the global health agenda, as outlined in the United Nations (UN) Sustainable Development Goals (SDGs) adopted in 2015. Indonesia’s National Health Insurance (Jaminan Kesehatan Nasional [JKN]), launched in 2014, has become Indonesia’s primary strategy for achieving UHC; however, JKN has been criticized for insufficiently addressing systemic inequities that put certain groups at a disadvantage [1].

Chronic diseases rank first in total claims submitted to *BPJS Kesehatan*, the national agency that manages the JKN [2]. Hypertension is the most prevalent condition, affecting more than 60% of individuals, followed by digestive diseases (44.5%) and arthritis (30.3%) [3]. Limited access to health facilities has reportedly reduced treatment adherence among patients with chronic conditions [4,5]. These challenges were further aggravated by the COVID-19 pandemic, which led to a decline in healthcare visits [6,7].

Disability is another critical dimension of health inequality. In Indonesia in 2020, the prevalence of disability was 1.34% among men and 1.53% among women, comprising about 3.9 million people nationwide [8]. At least 10.8% of adults with disabilities also live with chronic diseases [2,9,10], but people with disabilities have consistently reported lower satisfaction with JKN compared to the general population [9]. For those managing chronic conditions, disruption of services such as physical therapy during the pandemic increased the risk of deterioration, hospitalization, and long-term complications [11,12]. These disruptions have contributed to worsening health outcomes, increased hospitalizations, and long-term complications [13,14]. These effects were even more severe in underdeveloped areas with fragile infrastructure and chronic resource shortages [6,15].

The pandemic also amplified pre-existing structural inequalities embedded in the health system [16]. Women and people with disabilities were disproportionately affected due to multiple barriers, including gender norms, limited decision-making autonomy, and physical accessibility constraints [1,17]. Disability-based disparities in healthcare access are not limited to the pandemic but reflect persistent structural barriers in both routine and emergency contexts [18]. No prior study has examined how people with disabilities access healthcare under JKN.

This study evaluates the impact of the COVID-19 pandemic on disability-based disparities in chronic healthcare use under Indonesia’s UHC system. Although the pandemic is no longer an immediate crisis, its disruptive effects on continuity of care for chronic diseases remain a critical case for understanding systemic vulnerabilities in primary healthcare. This study examines whether these disparities are modified by gender and residence in underdeveloped regions and assesses the influence of demographic characteristics and JKN membership on healthcare visits during the pandemic.

## Method

### Data

We used the JKN administrative claims dataset, which covers 1% of JKN members. While secondary data may be limited by potential reporting bias, this dataset is mandatory for all health facilities and subject to verification and auditing processes by BPJS Kesehatan. This makes it the most comprehensive and standardized source of healthcare utilization data in Indonesia. The dataset contains health care history for all household members and representatives at the national, provincial, and district/city levels.

This study uses the 2020 primary healthcare subset, which includes all family members registered as JKN members as of 31 December 2020. This dataset also records all visits to the primary healthcare facility from 2019 to 2020 for each JKN member. Data on individual visits to primary health facilities are mapped monthly, covering six months before (September 2019 to February 2020) and after the Indonesian President declared the pandemic status on 2 March 2020.

### Sample

This study involved JKN members aged 19–65 with chronic diseases. Patients were categorized as having a chronic disease if they have a history of visits to a primary health facility with a diagnosis based on the International Classification of Diseases 10^th^ Revision codes for one or more of the following diseases: Diabetes Mellitus (E10–E14), Hypertension (I10–I15), Ischemic Heart Disease (I20–I25), Chronic Obstructive Pulmonary Disease (COPD) and Asthma (J40–J47), Chronic Kidney Disease (N18), Chronic Liver Disease (K70–K77), Cancer (C00–C97), and Chronic Mental Disorders (F20–F39). We acknowledge that other chronic conditions exist, but our inclusion was limited to those that align with the Ministry of Health’s priority programs in Indonesia [19]. Appendix 1 (Figure A1) displays a flow diagram of the sample used in this study. The dataset contained complete information for all variables included in the analysis. No observations were excluded due to missing data. Overall, this study included 108,762 JKN members with chronic diseases.

### Variables

The dependent variable was the frequency of visits to primary health facilities, defined as the monthly count of preventive or curative visits per individual during the six months before and after the pandemic was declared. *BPJS Kesehatan* recorded visits according to whether a patient was seen for preventive or curative services.

The primary independent variable was disability status. Individuals were classified as having a disability if they had received a qualifying diagnosis at least once during the study period. We followed the WHO manual on the International Classification of Functioning, Disability and Health (ICF), which states that impairments recorded as ICD-10 codes can be used as proxies for disability status in claims data [20]. Individuals were classified as having a disability if they received a diagnosis corresponding to at least one of the following: 1.) *Physical disability*: G80 (cerebral palsy), G83 (paralysis), Z89 (limb absence), M05–M19 (rheumatoid arthritis/arthrosis); 2.) *Sensory disability*: H54 (blindness/low vision), H90–H91 (hearing loss); 3.) *Intellectual disability*: F70–F79 (intellectual disability), F84 (pervasive developmental disorders); or 4.) *Mental disability*: F80–F89 (developmental disorders). Since JKN data does not directly record disability status, this operational definition provides a valid proxy while acknowledging that it may not fully capture the severity or functional limitations associated with these conditions.

Covariates included gender, age, JKN segmentation, residence in an underdeveloped district, household role, and ownership of the healthcare facility. The household role variable is defined to capture household members other than the head of household or children, which often include extended family members sharing a residence, a common custom in Indonesia. For categorical variables, the following reference categories were used: male for gender, young adults (19–25 years) for age group, non-worker for JKN segmentation, developed district for place of residence, household head for household role, and private provider for ownership of the healthcare facility. A detailed description of variables is listed in Appendix 2.

JKN segmentation refers to classifying memberships within Indonesia’s National Health Insurance system based on employment status, ability to pay premiums, and eligibility for government subsidies. There are four JKN membership segments: PBI, PBPU, PPU, and non-worker, all reflecting different funding sources and administrative pathways. PBI (Contribution Assistance Recipients) includes low-income individuals whose premiums are fully subsidized by the government. Informal workers, such as self-employed individuals, freelancers, farmers, and small business owners, are categorized as PBPU and are responsible for paying their premiums independently every month. Formal wage workers fall under the PPU category, with premiums jointly paid by employers and employees. Non-worker groups, including retirees, the elderly, and dependents without income, either pay their premiums or are covered by sponsors. In this study, the non-worker group is used as the reference category. Each individual registered as a JKN member must enroll with a designated facility, which serves as their primary healthcare provider and is responsible for delivering essential health services, managing chronic conditions, and coordinating referrals as necessary.

### Statistical analysis

This study employed a panel-based interrupted time series (ITS) design, analyzed using generalized estimating equations (GEE). Four models were tested sequentially: Model A served as the baseline ITS model, assessing changes in healthcare use before and after the onset of the COVID-19 pandemic. Model B extended Model A with an interaction between disability status and post-pandemic trends. Model C extended Model B by adding a disability-by-gender interaction to assess gender-based disparities among people with disabilities. Model D extended Model C by incorporating a three-way interaction between disability, gender, and residence in underdeveloped districts. All outcomes were defined as monthly counts of preventive or curative healthcare visits per individual. The main specification was:

$$\log\left(E\left[Y_{\mathrm{it}}\right]\right)=\beta_{0}+\beta_{1}.\mathrm{pos}t_{t}+\overset{5}{\underset{k=1}{\sum }}\beta_{2k}.\mathrm{time}\_\mathrm{afte}r_{\mathrm{kt}}+\beta_{3}.\mathrm{disabilit}y_{i}+X_{i}\gamma +\varepsilon_{\mathrm{it}}$$

where $Y_{\mathrm{it}}$ represents the outcome variable of individual *i* at month *t*, with the first month of observation being September 2019. $\mathrm{pos}t_{t}$ is the dummy variable for the period when the COVID-19 pandemic began in March 2020. $\mathrm{time}\_\mathrm{afte}r_{\mathrm{kt}}$ captures monthly trends in post-March 2020. $\mathrm{disabilit}y_{i}$ is the dummy variable of individual disability status (1 = has disability). $X_{i}$ represents the covariate variables used to adjust for characteristics such as gender, age, JKN segmentation, whether an individual lives in an underdeveloped district, household roles, ownership of the enrolled primary healthcare facility, and diagnosis of social problems. Diagnosis of social problems is defined using ICD-10 Z55–Z65 codes (e.g. problems related to education, employment, housing, family disruption, or poverty). These codes are recorded when healthcare providers identify social conditions that may affect a patient’s health status, and serve as a proxy for structural vulnerabilities in this study.

GEE models were estimated using a negative binomial distribution to account for overdispersion in the count data, confirmed through likelihood ratio tests of the dispersion parameter. A log-link function was employed to ensure positive predicted values. Coefficients are reported as Incidence Rate Ratios (IRRs) with 95% confidence intervals. An IRR > 1 was interpreted as increased utilization, while an IRR < 1 indicated decreased utilization. Statistical significance was defined at *p* < 0.05 (two-tailed). An exchangeable correlation structure was applied to account for within-individual correlation across repeated monthly observations. All covariates $X_{i}$ were simultaneously included in the regression models, and their estimated effects are presented alongside the main effects of disability, gender, and geography. We also compared the covariate estimates across Model D for preventive and curative outcomes to highlight differences in magnitude and significance.

All analyses were conducted using StataSE V.18.

## Results

### Sample characteristics

#### Entire analytic population

Table 1 summarizes the characteristics of 108,762 JKN members with chronic conditions. The weighted mean age was 48.3 years, and 61.2% were female. Only 1.2% resided in underdeveloped districts. Over half were principal household members (52.9%). Most enrollees (70.9%) were registered at government-owned primary care facilities. By JKN segmentation, the largest group was beneficiaries of government-subsidized insurance (PBI; 47.7%). Hypertension was the most common chronic disease (42.9%), and 10.4% had multimorbidity. A tiny proportion had a diagnosis of social problems (0.05%). Social problems diagnosis refers to the flag recorded in claims indicating a documented social problem.

**Table 1.** Sociodemographic characteristics of JKN members with chronic diseases at baseline by disability status and type of healthcare utilization (Indonesia, 2019–2020).

Variable	Total (*N* = 108,762)		No disability (*n* = 90,917)		With disability (*n* = 17,845)		Preventive care visits (*n* = 8,948)		Curative care visits (*n* = 32,677)	
	Unweighted No.	Weighted % or mean	Unweighted No.	Weighted % or mean	Unweighted No.	Weighted % or mean	Unweighted No.	Weighted % or mean	Unweighted No.	Weighted % or mean
Mean age (years)	48.27	48.3	47.87	48.0	50.32	50.0	48.8	48.9	49.5	49.5
Age group (%)										
– Young adult (19–25)	4,453	4.3	3,951	4.4	502	3.4	326	3.8	906	2.6
– Adult (26–44)	32,129	29.3	28,057	30.4	4,072	22.9	2,514	27.8	8,696	27.2
– Middle-age (45–59)	53,678	49.3	44,101	48.6	9,577	53.2	4,540	50.3	16,625	50.4
– Elderly (60–65)	18,502	17.2	14,808	16.6	3,694	20.4	1,568	18.1	6,450	19.7
Female (%)	64,141	61.2	53,472	60.9	10,669	62.6	5,561	62.8	19,813	63.2
Family role (%)										
– Principal	58,463	52.9	49,035	53.1	9,428	51.5	4,367	48.8	17,694	52.6
– Spouse	42,611	39.1	35,642	39.2	6,969	38.9	3,828	41.8	13,037	40.8
– Child	5,031	5.6	4,130	5.4	901	6.8	530	6.9	1,099	4.0
– Additional family member	2,657	2.4	2,110	2.3	547	2.9	223	2.6	847	2.6
Living in underdeveloped district (%)	1,998	1.2	1,722	1.2	276	1.0	73	0.4	445	0.9
Ownership of enrolled primary care facility: Government (%)	61,145	70.9	51,018	70.9	10,127	71.4	6,283	82.8	16,118	63.2
Has social problems diagnosis (%)	47	0.05	35	0.05	12	0.05	4	0.05	23	0.1
JKN segmentation (%)										
– Non-worker	6,106	4.8	4,815	4.5	1,291	6.0	463	4.3	2,359	6.2
– PBI (APBN/APBD)	35,378	47.7	29,474	47.6	5,904	48.3	4,518	63.8	9,060	40.1
– PBPU	27,118	17.7	22,339	17.2	4,779	20.3	1,309	10.5	8,773	21.4
– PPU	40,156	29.8	34,285	30.6	5,871	25.4	2,657	21.4	12,483	32.3
Chronic diseases (%)										
– Diabetes Mellitus	19,291	16.9	16,170	16.8	3,121	17.5	1,622	15.95	8,228	25.5
– Hypertension	47,542	42.9	39,056	42.5	8,486	45.6	4,177	44.52	17,072	52.3
– Ischemic Heart Disease	4,811	4.2	4,138	4.4	673	3.3	331	3.77	1,786	5.5
– COPD & Asthma	11,332	9.6	9,619	9.9	1,713	8.5	838	8.53	3,947	11.6
– Chronic Kidney Disease	1,595	1.5	1,455	1.6	140	0.7	104	1.05	580	2.0
– Chronic Liver Disease	793	0.7	684	0.7	109	0.8	60	0.54	262	0.8
– Cancer	3,179	2.6	2,899	2.8	280	1.4	184	2.23	929	2.7
– Chronic Mental Disorders	2,486	2.6	444	0.4	2,042	14.3	173	2.26	789	2.7
Has multimorbidity (%)	12,479	10.4	9,885	9.7	2,594	13.8	1,179	11.57	6,378	19.4
Type of disability (among disabled, %)							1,534	15.8	7,238	21.9
– Physical		–		–	14,944	81.1	1,315	13.0	6,251	18.6
– Sensory		–		–	1,220	6.3	103	1.1	502	1.4
– Intellectual		–		–	10	0.07	0	0.0	3	0.0
– Mental		–		–	2004	14.2	146	1.9	647	2.3
Multiple disability (among disabled, %)										
− 1 disability condition		–		–	17,513	98.3	7,414	84.2	7,074	21.4
− 2 disability conditions		–		–	331	1.6	1,504	15.5	163	0.4
− 3 disability conditions		–		–	1	0.0	30	0.25	1	0.0

*Note:* 1. Percentages are survey-weighted using the individual sampling weight (PSTV15); unweighted counts are shown in the ‘Unweighted N’ columns.

2. ‘Government’ = publicly owned facilities; ‘Private’ = non-government facilities.

3. ‘Social problems at diagnosis’ refers to cases with a recorded ICD-10 Z-code for social circumstances (e.g. inadequate housing, family conflict, or economic hardship).

4. Values for ‘Type of disability’ and ‘Multiple disability’ apply only to participants identified as having any disability.

#### Comparison by disability status

Overall, 15.5% had at least one disability; among these, physical disability predominated (81.1%) and almost all reported a single condition (98.3%). Compared with those without disabilities, individuals with disabilities were older on average (50.0 vs. 48.0 years) and were slightly more often female (62.6% vs. 60.9%). The proportion living in underdeveloped districts was similarly low in both groups (1.0% among people with disabilities vs. 1.2% among those without). Family role and ownership of the enrolled primary care facility showed little difference by disability status. By JKN segmentation, people with disabilities were more often non-workers. Regarding chronic conditions, people with disabilities had higher prevalences of hypertension, diabetes, and chronic mental disorders, and lower prevalences of ischemic heart disease, COPD/asthma, chronic kidney disease, and cancer. Multimorbidity was more frequent among people with disabilities, while the share with a social-problems diagnosis was similarly rare across groups.

#### Comparison by type of visit (preventive vs. curative)

Individuals with any preventive visit were more often enrolled in government primary care facilities (82.8%) and in the PBI segment (63.8%). In contrast, those with curative visits were more frequently PPU (32.3%) and less frequently PBI (40.1%), with lower enrollment at government facilities (63.2%). Residence in underdeveloped districts was uncommon in both groups. Importantly, sex and age distributions were broadly similar between preventive and curative users (female ≈63% in both; mean age 48.9 vs. 49.5 years). Chronic disease burden was consistently higher among curative users, including hypertension (52.3% vs. 44.5%), diabetes (25.5% vs. 16.0%), and multimorbidity (19.4% vs. 11.6%).

### Trends in healthcare utilization

The trends of the monthly average number of preventive and curative visits from September 2019 to August 2020 are depicted in Appendix 3 (Figure A2). Utilization was relatively stable before the pandemic (Sept 2019–Feb 2020). Both preventive and curative visits declined sharply after March 2020, reaching the lowest levels in April, before gradually recovering in subsequent months. Predicted values from the ITS models are presented in Appendix 4, with Figure A3 illustrating the predicted preventive visits and Figure A4 showing the predicted curative visits.

### Preventive care utilization

Table 2 presents the estimated effects of the COVID-19 pandemic on monthly preventive healthcare visits. In Model A, the onset of the pandemic was associated with a statistically significant decline in preventive care visits (IRR = 0.95, 95% CI: 0.92–0.98). Its time trends during the pandemic indicated a persistent reduction in preventive visits during the first five months following the onset. The most significant declines were observed in the first two months (IRR = 0.76 and 0.68). Model B showed that individuals with disabilities had slightly higher rates of preventive visits compared to those without disabilities (IRR ≈1.1). However, the statistically insignificant interactions between disability and time indicate a similar pattern of decline across both groups. In Model C, the interaction of disability and gender was also not significant, showing that gender did not moderate the effect of disability status on preventive care use during the pandemic. Finally, in Model D, the three-way interaction between disability, gender, and residence in underdeveloped districts was not statistically significant, showing that geographic disadvantage did not differentially affect preventive care across subgroups.

**Table 2.** Estimated effects of the COVID-19 pandemic on monthly preventive healthcare visits among JKN members with chronic diseases, Indonesia, 2019–2020.

Variable	Model A		Model B		Model C		Model D	
	IRR	95% CI	IRR	95% CI	IRR	95% CI	IRR	95% CI
COVID-19 pandemic	0.95***	0.92–0.98	0.95**	0.92–0.98	0.95**	0.92–0.98	0.95**	0.92–0.98
Time trend during the COVID-19 pandemic								
1	0.76***	0.71–0.81	0.76***	0.71–0.82	0.76***	0.71–0.82	0.76***	0.71–0.82
2	0.67***	0.63–0.72	0.68***	0.63–0.74	0.68***	0.63–0.74	0.68***	0.63–0.74
3	0.78***	0.74–0.82	0.78***	0.74–0.83	0.78***	0.74–0.83	0.78***	0.74–0.83
4	0.86***	0.82–0.90	0.87***	0.83–0.92	0.87***	0.83–0.92	0.87***	0.83–0.92
5	0.83***	0.79–0.88	0.83***	0.79–0.88	0.83***	0.79–0.88	0.83***	0.79–0.88
Has disability (vs no)	1.09*	1.00–1.18	1.09*	1.00–1.19	1.04	0.92–1.17	1.04	0.92–1.18
Female	0.98	0.92–1.05	0.98	0.92–1.05	0.97	0.90–1.04	0.97	0.90–1.04
Living in underdeveloped districts	0.32***	0.24–0.41	0.32***	0.24–0.41	0.32***	0.24–0.41	0.34****	0.19–0.60
Has disability × time trend during the COVID-19 pandemic								
1			0.93	0.82–1.05	0.93	0.82–1.05	0.93	0.82–1.05
2			0.88	0.76–1.00	0.88	0.76–1.00	0.88	0.76–1.00
3			0.98	0.87–1.11	0.98	0.87–1.11	0.98	0.87–1.11
4			0.93	0.83–1.04	0.93	0.83–1.04	0.93	0.83–1.04
5			1.02	0.91–1.14	1.02	0.91–1.14	1.02	0.91–1.14
Has disability × female					1.07	0.91–1.27	1.07	0.91–1.27
Female × living in underdeveloped district							0.82	0.42–1.57
Has disability × female × living in underdeveloped district							2.25	0.73–6.94
***Covariates***								
Age group								
Adult (26–44 years old)	0.98	0.85–1.13	0.98	0.85–1.13	0.98	0.85–1.13	0.98	0.85–1.13
Middle-age (45–59 years old)	1.07	0.91–1.26	1.07	0.91–1.26	1.07	0.91–1.26	1.07	0.91–1.26
Elderly (60–65 years old)	1.08	0.93–1.27	1.08	0.93–1.27	1.08	0.93–1.27	1.08	0.93–1.27
Family role								
Spouse	1.15***	1.07–1.23	1.15***	1.07–1.23	1.15***	1.07–1.23	1.15***	1.07–1.23
Child	1.18*	1.02–1.36	1.18*	1.02–1.36	1.18*	1.03–1.37	1.18*	1.03–1.37
Additional family member	0.95	0.82–1.10	0.94	0.81–1.10	0.94	0.81–1.10	0.94	0.81–1.10
Ownership of enrolled primary care facility: Government	1.66***	1.53–1.80	1.66***	1.53–1.80	1.66***	1.53–1.80	1.66***	1.53–1.80
Has diagnosed with social problems	0.81	0.34–1.93	0.81	0.34–1.91	0.81	0.34–1.91	0.81	0.34–1.91
JKN segmentation								
PBI	1.26**	1.08–1.48	1.26**	1.07–1.48	1.26**	1.07–1.48	1.26**	1.07–1.48
PBPU	0.78**	0.65–0.94	0.78*	0.65–0.94	0.78*	0.65–0.94	0.78*	0.65–0.94
PPU	0.94	0.81–1.10	0.94	0.80–1.10	0.94	0.80–1.10	0.94	0.80–1.10
N	108,758		108,758		108,758		108,758	

*p-value <0.05 **p-value <0.0 1 ***p-value <0.001.

*Note*: Estimates from GEE negative binomial models, reported as IRR (95% CI) with semirobust SE clustered by individual. Model A: baseline ITS (pre/post COVID-19). Model B: adds disability status. Model C: adds disability × gender interaction. Model D: adds disability × gender × underdeveloped district interaction.

After adjusting for covariates, preventive care use was higher among spouses (IRR = 1.15, 95% CI: 1.07–1.23) and children (IRR = 1.18, 95% CI: 1.03–1.37) compared with household heads. Enrollment in government-owned facilities (IRR = 1.66, 95% CI: 1.53–1.80) and in the government-subsidized insurance segment (PBI; IRR = 1.26, 95% CI: 1.07–1.48) was also associated with higher preventive use. In contrast, preventive use was lower among members in the PBPU segment (IRR = 0.78, 95% CI: 0.65–0.94) and those living in underdeveloped districts (IRR = 0.34, 95% CI: 0.19–0.60). No significant gender differences were observed.

### Curative care utilization

Table 3 presents the estimated effects of the COVID-19 pandemic on monthly curative healthcare visits among individuals with chronic conditions. Compared to preventive care visits, across all models, individuals with disabilities had significantly higher rates of curative visits compared to those without disabilities. In Model A, monthly curative visit rates sharply declined in the first two months after the COVID-19 pandemic onset, but overall use remained significantly higher during the pandemic (IRR = 1.05, 95% CI: 1.02–1.07). In Model B, a significant disability and time interaction indicated that individuals with disabilities had more curative visits during the pandemic compared to those without disabilities. Model C introduced the disability and gender interaction and showed that women with disabilities had significantly lower curative visits compared to men with disabilities (IRR = 0.92, 95% CI: 0.86–0.98). In Model D, the three-way interaction between disability, gender, and residence in underdeveloped districts was not significant.

**Table 3.** Estimated effects of the COVID-19 pandemic on monthly curative healthcare visits among JKN members with chronic diseases, Indonesia, 2019–2020.

Variable	Model A		Model B		Model C		Model D	
	IRR	95% CI	IRR	95% CI	IRR	95% CI	IRR	95% CI
COVID-19 pandemic	1.05***	1.02–1.07	1.05***	1.02–1.07	1.05***	1.02–1.07	1.05***	1.02–1.07
Time trend during the COVID-19 pandemic								
1	0.65***	0.63–0.67	0.64***	0.62–0.66	0.64***	0.62–0.66	0.64***	0.62–0.66
2	0.54***	0.52–0.56	0.53***	0.51–0.55	0.53***	0.51–0.55	0.53***	0.51–0.55
3	0.72***	0.70–0.74	0.70***	0.68–0.73	0.70***	0.68–0.73	0.70***	0.68–0.73
4	0.74***	0.71–0.76	0.72***	0.69–0.74	0.72***	0.69–0.74	0.72***	0.69–0.74
5	0.71***	0.69–0.73	0.71***	0.68–0.73	0.71***	0.68–0.73	0.71***	0.68–0.73
Has disability (vs no)	1.66***	1.61–1.72	1.71***	1.63–1.80	1.71***	1.63–1.80	1.72***	1.63–1.81
Female	1.09***	1.06–1.12	1.12***	1.07–1.14	1.12***	1.07–1.14	1.11***	1.07–1.14
Living in underdeveloped districts	0.76***	0.70–0.83	0.76***	0.70–0.83	0.76***	0.70–0.83	0.83*	0.70–0.99
Has disability × time trend during the COVID-19 pandemic								
1			1.08*	1.01–1.16	1.08*	1.01–1.16	1.08*	1.01–1.16
2			1.09*	1.01–1.18	1.09*	1.01–1.18	1.09*	1.01–1.18
3			1.10**	1.02–1.18	1.10**	1.02–1.18	1.10**	1.02–1.18
4			1.12***	1.05–1.19	1.12***	1.05–1.19	1.12***	1.05–1.19
5			1.01	0.95–1.08	1.01	0.95–1.08	1.01	0.95–1.08
Has disability × female			0.92*	0.86–0.98	0.92*	0.86–0.98	0.92*	0.86–0.98
Female × living in underdeveloped district						0.9		0.73–1.11
Has disability × female × living in underdeveloped district				1.16				0.76–1.77
***Covariates***								
Age group								
Adult (26–44 years old)	1.14**	1.05–1.25	1.14**	1.05–1.25	1.14**	1.05–1.25	1.14**	1.05–1.25
Middle-age (45–59 years old)	1.30***	1.19–1.42	1.30***	1.19–1.42	1.30***	1.19–1.42	1.30***	1.19–1.43
Elderly (60–65 years old)	1.46***	1.33–1.61	1.47***	1.33–1.61	1.46***	1.33–1.61	1.47***	1.33–1.61
Family role								
Spouse	0.99	0.96–1.02	0.99	0.96–1.02	0.99	0.96–1.02	0.99	0.96–1.02
Child	0.98	0.91–1.06	0.98	0.91–1.06	0.98	0.91–1.06	0.98	0.91–1.06
Additional family member	0.99	0.92–1.07	0.99	0.92–1.07	0.99	0.92–1.07	0.99	0.92–1.07
Ownership of enrolled primary care facility: Government	0.75***	0.73-.077	0.75***	0.73-.077	0.75***	0.73–0.77	0.75***	0.73–0.77
Has diagnosed with social problems	1.70***	1.29–2.23	1.70***	1.29–2.23	1.70***	1.29–2.23	1.70***	1.29–2.23
JKN segmentation								
PBI	0.80***	0.76–0.84	0.80***	0.76–0.84	0.80***	0.76–0.84	0.80***	0.76–0.84
PBPU	0.96	0.91–1.00	0.96	0.91–1.00	0.96	0.91–1.00	0.96	0.91–1.00
PPU	0.88***	0.84–0.93	0.88***	0.84–0.93	0.88***	0.84–0.93	0.88***	0.84–0.93
N	108,758		108,758		108,758		108,758	

*p-value <0.05 **p-value <0.01 ***p-value <0.001.

*Note*: Estimates from GEE negative binomial models, reported as IRR (95% CI) with semirobust SE clustered by individual. Model A: baseline ITS (pre/post COVID-19). Model B: adds disability status. Model C: adds disability × gender interaction. Model D: adds disability × gender × underdeveloped district interaction.

Curative service use was higher among women overall (IRR = 1.11, 95% CI: 1.07–1.14) and increased with age (e.g. elderly: IRR = 1.47, 95% CI: 1.33–1.61, compared to young adults). Curative visits were lower for those enrolled at government-owned facilities (IRR = 0.75, 95% CI: 0.73–0.77), for PBI (IRR = 0.80, 95% CI: 0.76–0.84) and PPU members (IRR = 0.88, 95% CI: 0.84–0.93), and for residents in underdeveloped districts (IRR = 0.83, 95% CI: 0.70–0.99). Having a diagnosis of social problems was associated with greater curative use (IRR = 1.70, 95% CI: 1.29–2.23).

## Discussion

This study evaluated the COVID-19 pandemic’s impact on disability-based disparities in healthcare use under Indonesia’s universal health coverage (JKN). Three key findings emerged. First, preventive and curative service use declined sharply at the pandemic onset, though curative services rebounded faster. Second, persons with disabilities consistently had higher curative utilization than those without disabilities, while no significant differences were observed in preventive services. Third, disparities were moderated by gender but not by residence in underdeveloped districts, while family structure and JKN segmentation also shaped patterns of healthcare use.

### Preventive and curative services during the pandemic

The COVID-19 pandemic led to a reduction in preventive and curative health visits for all patients with chronic conditions, regardless of their disability status. Similar trends have been reported in Indonesia and other lower- to middle-income countries (LMICs), where patients with hypertension and diabetes faced significant disruptions in follow-up care and treatment adherence [21–23]. In high-income countries with stronger integration of telemedicine, preventive and mental health services also suffered significant declines [24–26]. In our study, however, the higher curative utilization among people with disabilities may reflect the compounded effect of disability and chronic conditions, which often necessitate urgent treatment.

Preventive visits among people with disabilities were slightly higher than among those without disabilities, but both groups experienced similar declines during the pandemic. By contrast, people with disabilities consistently showed significantly higher rates of curative visits with a marked increase during the pandemic. In Indonesia, curative treatment for chronic diseases was classified as an essential health service and continued officially. Yet, group-based physical sessions under PROLANIS *(Program Pengelolaan Penyakit Kronis, or Chronic Disease Management)*, a program specifically designed for JKN enrollees with chronic conditions, were suspended. At the same time, telemedicine was increasingly promoted as a substitute for routine follow-up and was recorded as a preventive visit. These adjustments, combined with more limited physical mobility, may have left unmet preventive needs that led to a greater reliance on curative services among people with disabilities.

The COVID-19 pandemic caused a significant systemic disruption to health systems worldwide [16]. Control measures, such as lockdowns and physical distancing, served as an institutional barrier to healthcare access [27]. The pandemic also created a collective fear of disease transmission among high-risk populations, including many patients with chronic conditions [28,29]. Our results showed a pattern of higher health service utilization among people with disabilities, consistent with their vulnerable position within the social structure. Compared to those without disabilities, patients with disabilities had similar access to preventive visits but relied more heavily on curative services during the pandemic. This suggests that their chronic disease needs were less likely to be met through preventive care and more often escalated into urgent curative care. As chronic patients, their urgent medical needs forced them to continue visiting clinics despite systemic disruptions, reflecting unmet needs rather than system effectiveness in protecting vulnerable populations during a crisis.

### Structural barriers for people with disability accessing healthcare services

Our results suggest that gender functioned as a structural moderator for the disabled population’s access to curative health services during the pandemic. Women with disabilities had significantly lower rates of curative visits compared to their male counterparts, despite no significant differences in preventive care. This pattern suggests that gender-based disparities are particularly pronounced in types of care that require greater resources, mobility, and decision-making autonomy, such as curative services [30–32]. In Indonesia, labor force participation among women is substantially lower than among men (13.93% vs. 30.46%), and participation is even lower among women with disabilities [6]. Limited income opportunities make women more vulnerable to the indirect costs of healthcare, such as transportation, foregone wages, and out-of-pocket expenses that JKN does not cover. During the pandemic, as observed in India, limited mobility and reliance on household decision-makers may have further exacerbated these disparities [33]. Spousal permission is often required for women to seek care, and more serious decisions, such as referrals, are typically discussed collectively within the extended family [34,35]. This reflects Indonesia’s multigenerational household composition, where intra-household hierarchies can delay access for those in peripheral roles within the family [36]. Such family norms intersect with gender, creating structural barriers to healthcare.

Family structure also shaped healthcare use. Descriptively, both preventive and curative visits were most common among immediate family members (spouses and children) compared to the head of household, while other household members had the lowest utilization. However, in adjusted analyses, preventive visits were significantly more common among spouses and children compared to the household head, suggesting that intra-household dynamics can facilitate preventive care for these groups. It is important to note that the majority of principal household members were male (around 65.2%), which may partly explain this pattern. The higher preventive utilization among spouses and children may reflect sex-related differences in health-seeking behavior. Women generally exhibit greater preventive health-seeking tendencies, and children are more likely to visit healthcare facilities for mandatory immunization and routine checkups. By contrast, no significant differences were observed by family role for curative services. This finding aligns with previous Indonesian studies, which show that family norms significantly influence healthcare decision-making [34,35].

JKN segmentation and geographic disadvantage also influenced healthcare use. Government-subsidized (PBI) members had higher preventive but lower curative utilization, which may reflect stronger links with community-based promotive programs but financial and logistical barriers to curative services. Enrollment in government-owned facilities was associated with greater preventive but lower curative care, possibly reflecting differences in provider capacity and referral practices. Similar findings in other Asia-Pacific countries show that residing in underdeveloped districts reduces both preventive and curative visits across all subgroups [16]. Hence, geographic disadvantage functions as a broad structural barrier that is not necessarily associated with disability or gender.

### Strengths and limitations of the study

This study is one of the first studies to examine disability-based disparities in healthcare under Indonesia’s UHC system during a crisis. It uses a large, nationally representative dataset of JKN enrollees and applies a panel-based interrupted time series design for causal inference. However, some methodological limitations should be taken into consideration.

First, because we used administrative claims data, disability and chronic disease status were defined based on ICD-10 diagnosis codes, which may have introduced misclassification and underreporting. While this approach adheres to the WHO International Classification of Functioning, Disability and Health (ICF) framework, the absence of functional assessments, such as the Activities of Daily Living (ADL), prevents the severity and impact of disabilities from being fully captured. Second, this study used a facility-based dataset, which may affect internal validity, capturing individuals who accessed healthcare services and excluding those who faced barriers to care and did not seek treatment. As a result, our findings may underestimate the extent of access disparities, particularly among marginalized populations. Third, the study period captures the early stage of the pandemic (2019–2020), and results may not fully reflect longer-term patterns.

Although formal sensitivity analyses were not conducted, model diagnostics were performed to ensure robustness of the estimates. Future work could explore the sensitivity of findings to alternate time windows, disability categorizations, and model structures. Future analysis should complement facility-based analyses with a population-based survey that includes functional measures of disability over a longer time frame to capture the long-term effects of the pandemic disruption on healthcare access and use.

## Conclusions

This study revealed that Indonesia’s reliance on telemedicine to maintain preventive care during the COVID-19 pandemic did not adequately serve people with disabilities, who continued to depend disproportionately on curative visits to meet their health needs. These patterns suggest that the health system was less responsive to the preventive care needs of persons with disabilities, reflecting structural gaps in accessibility and continuity of chronic disease management. The intersection of disability, gender, and socioeconomic status further shaped healthcare use: women with disabilities faced compounded barriers linked to limited mobility, economic dependence, and household decision-making norms.

To advance equity under universal health coverage, Indonesia’s National Health Insurance (JKN) must go beyond financial protection toward inclusive service delivery. Integrating Gender Equality, Disability, and Social Inclusion (GEDSI) into JKN monitoring and crisis response – especially within digital health and chronic disease programs – is essential. Targeted policies that ensure accessible telemedicine, strengthen preventive care, and address intersecting vulnerabilities will be critical to achieving equitable and resilient healthcare for all Indonesians.

## Supplementary Material

Reporting Guideline.docx

Appendix file_author.docx

## Data Availability

This study is based on secondary analysis of the Indonesian National Health Insurance (JKN) sample dataset provided by BPJS Kesehatan. The data are publicly available upon formal request and approval through the official BPJS Kesehatan data access portal (https://data.bpjs-kesehatan.go.id). The authors did not create or collect new data.
