# Supplementary material for: Disability-based disparities under universal health coverage among chronically ill adults during the COVID-19 pandemic in Indonesia: an interrupted time series analysis
Source: Glob Health Action. 2025 Nov 7;18(1):2581946. doi: 10.1080/16549716.2025.2581946 (PMC12599157; doi:10.1080/16549716.2025.2581946)
Supplement: Appendix file_author.docx [file ZGHA_A_2581946_SM1538.docx]

**Appendix 1. Flow diagram of the study sample**

108,762 patients

5,071,123 visits:

- 2,726,234 visits in 2019
- 2,344,889 visits in 2020

884,917 patients:

- 695,145 patients in 2019
- 189,772 patients in 2020

661,292 patients

223,625 patients dropped due to time visits not between Sep 2019 to Aug 2020

552,530 patients dropped due to not satisfying the sample criteria:

1. Has chronic disease
2. Aged 19-65 at Sep 2019

Figure 1. Flow diagram of the study sample

Note:

We define the onset of the pandemic as March 2, 2020, when the government announced the first confirmed case.

**Appendix 2. List of variables**

| Variable | Type of variables | Coding |
| --- | --- | --- |
| ***Covariates*** |  |  |
| Female | Binary | 0 : Male  1 : Female |
| Age (years) | Continuous |  |
| JKN segmentation | Categorical | 0 : Non-Worker  1 : PBI: Penerima Bantuan Iuran (government-subsidized)  2 : PBPU: Pekerja Bukan Penerima Upah (self-employed)  3 : PPU: Pekerja Penerima Upah (formal wage workers) |
| Live in underdeveloped district | Binary | 0 : not in underdeveloped district  1 : in underdeveloped district |
| Family role | Categorical | 0 : Principal  1 : Spouse (wife or husband)  2 : Children  3 : Additional family member |
| Enrolled to government-owned PHC | Binary | 0 : Private-owned PHC  1 : Government-owned PHC |
| Has disability  *at least has one of disability condition below:*   - Physical disabilities (ICD10: G80, G83, Z89, Z89, M05 M19) - Sensory disabilities (ICD10: H54, H90, H91) - Intellectual disabilities (ICD10: F70, F79, F84, F84) - Mental disabilities (ICD10: F20, F29, F30, F39, F80, F89) | Binary | 0 : Do not have any disability  1 : Have at least one disability condition (physical, sensory, intellectual, mental) |
| Has diagnosed with social problem  *Identified with ICD10* ***Z55-Z65*** | Binary | 0 : No  1 : Yes |
|  |  |  |
| ***Outcomes*** |  |  |
| Healthy visit (for preventive care) | Discrete |  |
| Sick visit (for curative care) | Discrete |  |
|  |  |  |
| ***ITS time variables*** |  |  |
| Time |  | Sep 2019=1 to Aug 2020=12 |
| post |  | 0 = Before March 2020  1= March to Aug 2020 |
| time_after |  | Before March 2020=0  March 2020=1  April 2020= 2  May 2020=3  June 2020=4  July 2020=5  Aug 2020=6 |

**Appendix 3. The trend of preventive and curative visits before and during COVID-19, September 2019–August 2020**

|  | Preventive Visit | | | Curative Visit | | |
| --- | --- | --- | --- | --- | --- | --- |
| Month | Mean | SE | Percentage Change | Mean | SE | Percentage Change |
| Sep-19 | 0.1767 | 0.001 |  | 0.1829 | 0.001 |  |
| Oct-19 | 0.1794 | 0.001 | 1.53% | 0.1928 | 0.001 | 5.41% |
| Nov-19 | 0.1742 | 0.001 | -2.90% | 0.1826 | 0.001 | -5.29% |
| Dec-19 | 0.1715 | 0.001 | -1.55% | 0.1837 | 0.001 | 0.60% |
| Jan-20 | 0.1773 | 0.001 | 3.38% | 0.2108 | 0.001 | 14.75% |
| Feb-20 | 0.1777 | 0.001 | 0.23% | 0.2059 | 0.001 | -2.32% |
| **Mar-20** | 0.1713 | 0.001 | -3.60% | 0.2016 | 0.001 | -2.09% |
| Apr-20 | 0.1275 | 0.001 | -25.57% | 0.12 | 0 | -40.48% |
| May-20 | 0.112 | 0.001 | -12.16% | 0.0962 | 0.001 | -19.83% |
| Jun-20 | 0.1283 | 0.001 | 14.55% | 0.1324 | 0.001 | 37.63% |
| Jul-20 | 0.1423 | 0.001 | 10.91% | 0.1354 | 0.001 | 2.27% |
| Aug-20 | 0.1417 | 0.001 | -0.42% | 0.1294 | 0.001 | -4.43% |

Figure 2. The trend of preventive and curative visits before and during COVID-19, September 2019 – August 2020

**Appendix 4. Predicted preventive and curative care visits**

Figure 3. Predicted preventive care visits

Figure 4. Predicted curative care visit
